# The relationship between APOE genotype and dementia varies by genetic ancestry and race/ethnicity

**DOI:** 10.1002/alz.093068

**Published:** 2025-01-09

**Authors:** Marian J Gilmore, Alexandra M Varga, Catherine Schaefer, Yambazi Banda, Thomas J. Hoffmann, M. Maria Glymour, Sheila A Weinmann, Hélène Choquet, Ning Smith, Paola Gilsanz, Stefan Massimino, Willa D Brenowitz

**Affiliations:** ^1^ Kaiser Permanente Center for Health Research, Portland, OR USA; ^2^ Kaiser Permanente Northern California Division of Research, Oakland, CA USA; ^3^ Kaiser Permanente Research Bank, Oakland, CA USA; ^4^ University of California San Francisco, Department of Epidemiology and Biostatistics, San Francisco, CA USA; ^5^ Boston University School of Public Health, Boston, MA USA; ^6^ Kaiser Permanente Division of Research, Oakland, CA USA

## Abstract

**Background:**

Some evidence suggests the effect of APOE genotype on dementia risk may vary by race/ethnicity and genetic ancestral background. We evaluated APOE genotype and associated dementia risk by genetic ancestry, with a focus on expanding research among participants with non‐European ancestry.

**Method:**

The Kaiser Permanente Research Bank (KPRB), one of the largest biobanks in the US, enrolled members of an integrated health system, collected genotyping and health surveys and link to electronic health records (EHR). Genetic ancestry proportions (range 0‐1) were estimated using the program ADMIXTURE. First diagnosis of all‐cause dementia was collected from EHR over a mean follow‐up time of 14.6 years. Logistic regressions evaluated associations of APOE e4 and e2 allele status with dementia adjusted for sex and age. We tested interactions between APOE and race/ethnicity (from self‐report and/or EHR) and separately APOE and genetic ancestry.

**Result:**

Preliminary analyses included 180,345 participants (mean age at consent 56.17 years), including 17,356 Asian, 11,595 Black, 926 Hawaiian/Pacific Islander, 22,663 Hispanic, 3,714 multiracial, and 498 Native American/Alaska Native participants. APOE e4 allele prevalence was highest among Black participants (34%) and lowest among Asian participants (17%). APOE e2 allele prevalence was highest among Black participants (17%) and lowest among Hispanic (8%) and Native American/Alaskan Native (9%) participants. APOE e4 allele (but not APOE e2) significantly interacted with both race/ethnicity and genetic ancestry, although associations were elevated among all groups. The association of APOE e4 with dementia diagnosis (n = 8,288) was strongest among White participants (OR: 1.67; 95%CI:1.59,1.75) and was significantly lower among Black (OR interaction: 0.83; 95%CI:0.69,0.99) and Hispanic participants (OR interaction:0.79; 95%CI:0.65,0.95). Similarly, the association of APOE e4 with dementia increased with higher proportion of European ancestry (OR interaction:1.30, 95%CI:1.13,1.49). The association of APOE e4 allele with dementia was reduced with higher proportion African ancestry (OR interaction:0.71, 95%CI: 0.57,0.90), and Native American ancestry (OR interaction: 0.52, 95%CI: 0.30,0.91).

**Conclusion:**

In preliminary analyses, we found APOE e4 allele association with dementia differed by European, African, and American genetic ancestries, which correlated with differences by race/ethnicity. Our findings suggest genetic ancestral background is important when utilizing APOE genotype in determining risk for dementia.

